# Leaf-DETR: Progressive adaptive network with lower matching cost for dense leaves detection

**DOI:** 10.1016/j.plaphe.2026.100182

**Published:** 2026-02-17

**Authors:** Xiaoyang Wan, Yuxiang Wang, Xinyu Dong, Lufu Qin, Xixuan Luo, Peijia Yu, Liang Lei, Chao Sun, Asa Salgadoe, Jingjing Jiang, Qi Wang

**Affiliations:** a State Key Laboratory of Public Big Data, College of Computer Science and Technology, Guizhou University, China; b The School of Physics & Optoelectronic Engineering, Guangdong University of Technology, Guangzhou, 510006, China; c Suninfinit Agriculture Group Co., Ltd, Shenzhen, China; d Department of Horticulture and Landscape Gardening, Faculty of Agriculture and Plantation Management, Wayamba University, Sri Lanka

**Keywords:** Leaf monitoring, Dense object detection, Progressive fusion, Query optimization strategy

## Abstract

Leaves are central indicators of photosynthesis and plant growth status, and their precise monitoring is crucial for smart agriculture. Dense leaf detection, as a foundation for leaf morphology analysis, must address challenges such as occlusion and overlap, directly enabling key tasks including phenotypic trait extraction, disease identification, and yield estimation. Leaves are the most important plant organs, and monitoring leaves is a crucial aspect of crop surveillance. Dense leaf detection plays an important role as a fundamental technology for leaf monitoring. Existing dense leaf detection methods rely on traditional modular detectors and generic feature extraction, lacking designs tailored to real-world dense leaf scenarios. The methods for dense leaf detection generally use traditional modular detectors and general feature extraction techniques, without designing methods specifically for dense leaves in reality. In detail, in complex field scenarios, it still faces challenges like incomplete individual feature extraction due to high leaf overlap and difficult network convergence caused by excessive leaf density. To this end, we propose the Leaf-DETR framework, which effectively addresses these challenges through the Progressive Feature Fusion Pyramid Network (P-FPN) and the Crowded Query Refinement Strategy (CQR). First, we construct the largest dense leaf detection dataset to date, containing 1696 images and 85,375 annotation boxes. Second, P-FPN alleviates the feature confusion problem of overlapping leaves through the multi-stage fusion of features and the Adaptive Feature Aggregation module (AFA), enhancing the interaction between low-level details and high-level semantics. Third, the CQR strategy significantly reduces the matching cost of crowded candidate boxes and improves the network convergence efficiency by culling a crowded query method and introducing a one-to-many matching mechanism. Finally, experimental results show that Leaf-DETR improves mAP@50 by 1% and AR@300 by 1.4% over the baseline model on our self-constructed dataset, outperforming existing detection methods. Furthermore, the model exhibits extremely fast training convergence and demonstrates strong generalization capability on both field-collected monitoring images and other staple crops, fully highlighting its practical value in complex agricultural scenarios. Finally, experiments show that Leaf-DETR outperforms existing detection methods on the self-built dataset and demonstrates good performance generalization in monitoring collected images, as well as for other staple food crops, which verifies its practicality in complex agricultural scenarios.

The code and detailed information are available at http://leafdetr.samlab.cn.

## 1 Introduction

**Fig. 1:**
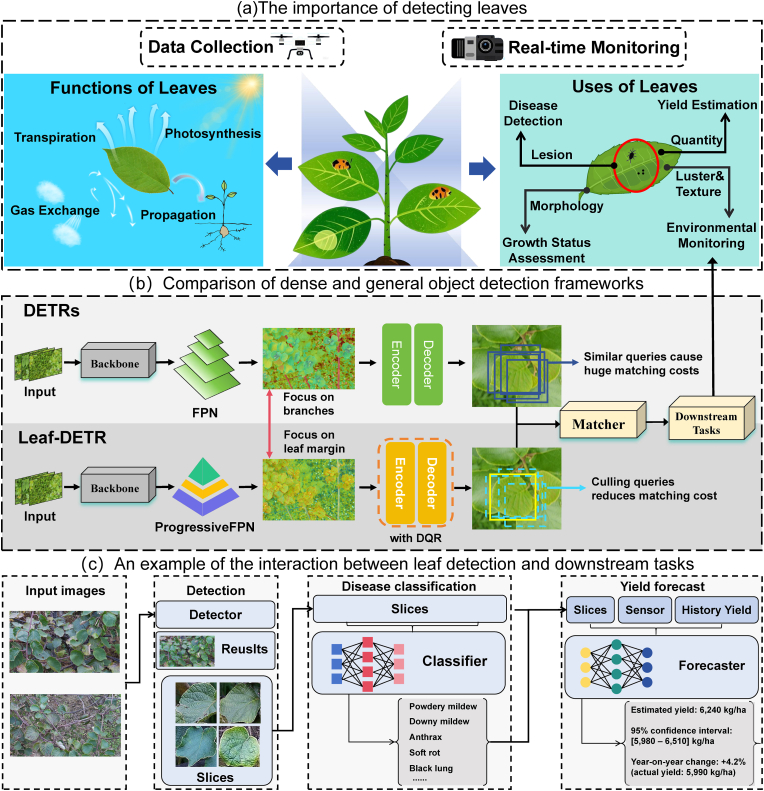
Motivation of leaf-deter. (a) The importance of leaves and the role of detecting leaves. (b) Differences in the DETRs architecture, Leaf-DETR has higher attention to the leaf edges and lower matching cost. (c) An example of linking object detection with downstream tasks, including disease classification and yield prediction.

Crop growth monitoring is crucial for sustainable agricultural production, as it enables the timely detection and resolution of issues affecting crop health and yield, thereby optimizing resource allocation and enhancing production efficiency [1]. Leaves, being the primary photosynthetic organs, play a pivotal role in this ecosystem by absorbing sunlight, carbon dioxide, and water to synthesize organic compounds essential for plant development [2]. Consequently, monitoring leaf characteristics is indispensable for assessing the overall crop health status [3]. As shown in Fig. 1, by combining advanced object detection techniques with downstream tasks to monitor leaves, it is possible to provide comprehensive information regarding leaf distribution, health status, and growth trajectories in complex agricultural environments where there is widespread leaf overlap and plant structures form complex visual patterns [4]. Among them, the dense object detection technology is extremely important for achieving the detection of dense leaves, as the foundation for downstream applications. For example, segmenting single leaf images through object detection can effectively facilitate plant disease identification [5], and these images can be further combined with prior knowledge to assess plant disease levels [6,7]; these methods can all effectively protect food security [8].

Specifically, dense object detection technology has already demonstrated great potential for application in the fields of computer vision and smart agriculture [9]. In the field of computer vision, researchers have adopted some novel methods to handle dense object detection tasks. Oksuz et al. [10] propose the MoCAE architecture, which calibrates and aggregates the prediction weights of different expert models through posterior learning, enabling the integrated model to improve its accuracy on the dense satellite image dataset DOTA [11]. Zheng et al. [12] design a progressive prediction method, which models the relationships between higher-quality queries and other queries, and refines noisy queries, thereby improving the performance in crowded scenes and enhancing the performance of the dense crowd detection task CrowdHuman [13]. In the agricultural field, Liu et al. [14] equip the YOLOv5 model with BiFPN [15], SimAM [16], and four detection heads to enhance the model's detection accuracy for dense corn tassels. Especially in the leaf detection task, Wang et al. [17] integrate CBAM [18] and DIoU-NMS [19] into Faster R-CNN to improve the RPN network [20]. The introduction of DIoU-NMS makes the RPN network more sensitive to the scale of anchor boxes and the offset of center points, improving the quality of proposal anchors and achieving good detection results on the denser outdoor sweet potato leaf detection dataset. Overall, these studies have all promoted the further development of dense leaf detection. However, these methods primarily focus on improving bounding box localization accuracy and do not address the challenges posed by complex leaf stacking and high inter-leaf similarity, indicating that detection approaches tailored for densely arranged leaves still require further exploration.

Specifically, there are three challenges in the field of dense leaf detection. Despite the significant progress made in these studies, there are still three problems in the dense leaf detection area. First, limited by the dense leaf datasets, researchers find it difficult to obtain sufficient general knowledge for small-scale fine-tuning work, which leads to limitations in practical applications [21]. Second, given the inherent complexity of dense leaf detection in field environments, existing methods often fail to address two challenges in the feature-extracting aspect [22]. In detail, the high visual similarity between foliage and background elements [23], and the difficulty in differentiating overlapping plant structures with analogous textures [24], collectively result in a substantial loss of fine-grained information during the hierarchical visual processing pipeline. Thirdly, the main challenge in dense leaf detection lies in the crowded anchor boxes resulting from the structural feature of a high overlapping rate between adjacent leaves [25]. This inherent feature leads to the fact that the surface leaves can be reliably identified [26], yet the partially occluded leaves are often missed by the detection system. Overall, these compounded factors collectively impose greater technical difficulties in dense foliage analysis compared to standard scenarios, significantly elevating the complexity of the detection task.

To solve these challenges, first, we propose a high-quality dense leaf detection dataset to provide a foundation for leaf monitoring tasks to overcome the lack of high-quality datasets for dense leaf detection. Second, for the phenomenon of confusion in feature extraction caused by leaf overlap, we refer to the practice of stacking modules and use a progressive construction method for feature decoupling. Finally, for the issue of high costs associated with the Hungarian Matching Algorithm^1^ and poor network convergence due to overlapping competition among crowded candidate boxes, we adopt the crowded candidate box culling method to reduce the burden of network training and improve performance.

In detail, we contribute a high-resolution dense kiwifruit leaf detection dataset featuring high-quality leaf edges and comprehensive surface characteristics, providing valuable resources for both immediate applications and transfer learning to related agricultural tasks. Moreover, we design Leaf-DETR, a dense leaf object detection framework incorporating a Progressive Feature Fusion Pyramid Network (P-FPN) and an efficient Crowded Query Refinement (CQR) strategy. Our P-FPN continuously integrates low-level features with high-level features during the upsampling process, ensuring that distinctive characteristics remain preserved throughout feature extraction. This approach prevents feature confusion typically encountered in dense scenes with visually similar objects [27]. Additionally, the proposed CQR strategy progressively reduces query complexity during the learning process, thereby enhancing the network's training efficiency and stability. This strategy operates in parallel with the original model branch, generating supplementary queries that facilitate more effective network learning while maintaining computational efficiency. Experiments show that Leaf-DETR has superior performance in the task of dense leaf object detection and also demonstrates good generalization performance in monitoring and collecting images as well as detecting other staple food crops. This study makes the following contributions:

• The largest dataset for dense leaf detection has been created, which includes 1696 images of dense kiwifruit leaves, with a total of 85,375 target objects. Each image has a high resolution of six million pixels and contains detailed phenotypic characteristics of kiwifruit leaves.
• A dense leaf detection framework Leaf-DETR is proposed. This framework is equipped with a Progressive Feature Pyramid Network, which is used to solve the problem of feature confusion caused by the mutual occlusion of dense leaves. Meanwhile, a Crowded Query Refinement strategy is adopted to overcome the challenge of difficult convergence that the network faces during the training of dense object tasks.
• The proposed dense leaf detection framework outperforms other models in terms of performance on the dense kiwifruit leaf detection dataset, and it also demonstrates excellent generalization performance on surveillance images and other staple food crops.

## 2 Materials and methods

### 2.1 Dataset

Although leaf monitoring is of great significance, currently there are relatively few object detection datasets in the agricultural field. These datasets mainly focus on leaf disease detection and tassel detection, and there is a lack of dense leaf object detection datasets that have greater application value. Current agricultural leaf detection datasets, such as PlantDoc [28] and PlantVillage [29], mainly focus on single-leaf disease detection. These datasets have significant limitations in practical field applications as they are insufficiently annotated for scenes of dense and overlapping leaves. Although existing tree-leaf datasets [30] and sweet potato leaf datasets [17] do address the situation of dense leaves to some extent, they often only annotate prominent and complete leaves, ignoring occluded leaves, or the scenes are limited to laboratories, thus restricting their practicality in complex agricultural environments. Therefore, we aim to construct a dataset with dense and highly overlapping leaves, similar to real-world scenarios, to address the issue of insufficient model applicability caused by current datasets.

#### 2.1.1 Equipment and crop selection

**Fig. 2:**
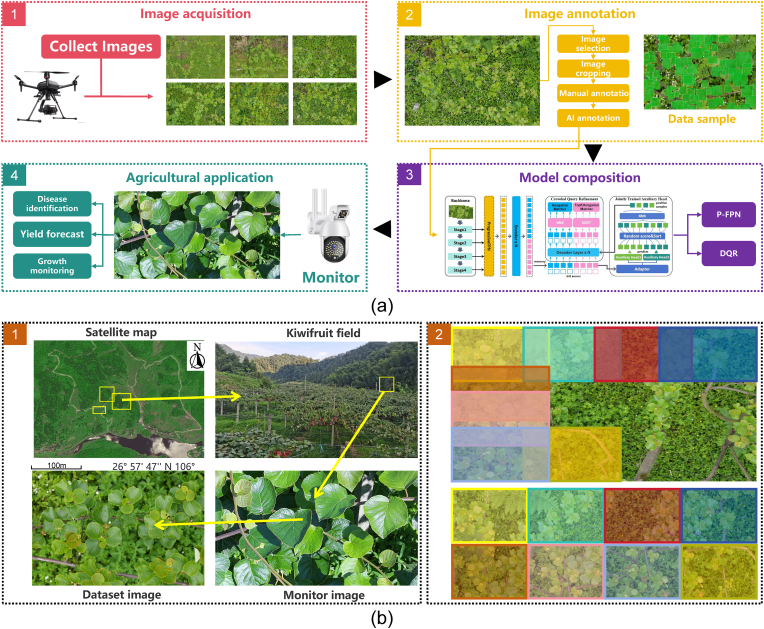
Schematic diagram of the kiwifruit leaf data pipeline and applications. (a-1) Drone data collection. (a-2) Image annotation. (a-3) Model architecture. (a-4) Agricultural applications. (b-1) Data collection locations. (b-2) Schematic diagram of image cropping methods.

Given that kiwifruit disease manifestation predominantly occurs in leaves [31] and considering the scaffold-based growth pattern of kiwifruit vines [32], we adopt kiwifruit leaves as the collection objects and employ Unmanned Aerial Vehicle (UAV) technology to obtain phenotypic data. This approach offers several advantages: (1) Superior Mobility: UAVs can efficiently cover extensive field areas in minimal time, enabling comprehensive data collection [33]. (2) Enhanced Field of View: The aerial perspective provides optimal coverage of the canopy structure [34]. (3) High-quality Texture Capture: Compared to handheld devices, UAV-based imaging can capture clear textural features of leaves’ surfaces [35], minimize interference from weeds and vines, and collect pure kiwifruit leaf image data with minimal background noise [36]. Our data flow is shown in Fig. 2(a). Using the DJI Mavic Air 2 drone as a carrier, the maximum endurance time is 45 min, the longest endurance distance is 23 km, and the maximum flight speed is 5 m/s. Equipped with a SONY A7R5 model camera, featuring a SONY SEL70200GM2 visible light lens, and using the DJI Rhino3 stabilizer for fixation and angle control.

#### 2.1.2 Data collection

In order to improve the quality of the dataset, we have customized the following collection methods and processing procedures.

1. **Collection Time and Location.** The collection time is from June 15th to September 15th, at the Jiuchang Kiwifruit Base in Xiuwen County, Guiyang City, Guizhou Province, China. The kiwifruit leaves are complete and plump, facing toward the sky, which is consistent with the perspective of the drone. The leaf surface has obvious textures, with clear edges and a high density, making them suitable for creating a dataset. The camera is arranged in the field, with a height of 4 m and a distance of 1.8 m from the leaf plane. There are twelve circular scanning positions, and the specific collection locations are shown in Figs. 2(b-1).
2. **Settings and Operation Methods of UAV Data Collection.** The captured images are in JPEG format with a size of sixty-one million pixels. The drone's flight speed and direction, as well as the camera's shooting angle, are manually controlled, with the camera's vertical angle to the ground being 80-90°. The drone has precise GPS positioning capabilities, allowing detailed recording of each image's location and altitude. The drone's flight altitude is approximately 16 m, with an aperture value of 5.6, exposure time of 1/125 s, ISO 80, focal length of 200 mm, continuous flight for 600 s, and a flight speed of 1 m/s. The drone performs a grid scan of the fields in the valley [37]. It first flies from the left to the right, then moves forward a certain distance, and returns from the right to the left, repeating this process until the designated area is fully covered [38].
3. **Image Screening Criteria.** Image screening criteria collected by drones include: excluding images without kiwi leaves; filtering out images with fewer than 200 leaves visually; removing images containing interfering elements such as roads or people; eliminating images where leaf distribution is too scattered at the edges and the center area has no leaves.
4. **Image Cutting Criteria.** As shown in Fig. 2(b-2), the sliding window method was used for image cutting. Based on the camera pixels and the distribution of anchor box, the window width and height are 9504∗6336, with a step size of 5000, other potential issues are discussed in the Supplementary S.3. If the distribution of image blades in the window was not satisfied or the number of blades was less than 50, they were removed.
5. **Image Annotation Criteria.** Half of the images were randomly selected for manual annotation, and then all were used as the training set to train the annotation model, which is based on Deformable-DETR. Its evaluation metrics are provided in Table 2, and further model details can be found in the Supplementary S.4. The remaining half of the images were annotated by the model, and the annotation box was manually fine-adjusted to ensure quality. The marking box only marks the visible parts of the blade, and parts lower than 1/3 of the blade are not marked. Both mature and primary leaves are marked, while leaves that are not expanded are not marked.

#### 2.1.3 Dataset statistics

As shown in Table 1, our dense kiwifruit leaf object detection dataset contains 1696 images with 85375 annotation boxes, on average of 50 detectable objects per sample. Compared with agricultural dense detection datasets [39,40], it has a higher greater density, and it is the largest dense leaf target detection dataset in the agricultural field. Compared to the traditional human detection datasets [13,41], the number of included instances per image in this dataset is much higher.

**Table 1:** Comparison of object detection datasets across different fields.

Field	Dataset	images	boxes	boxes/image(density)
Computer Vision	COCOPersons [42]	64115	267252	4.01
	CrowdHuman [13]	15000	339565	22.64
	WiderPerson [41]	13382	273475	20.43
Agriculture Field	GWHD2020 [43]	4700	190000	40.42
	GWHD2021 [39]	6422	271553	42.28
	MaizeTassel [40]	361	13564	37.57
	PlantDoc [28]	2581	8921	3.45
	KiwiFruitLeaf (ours)	1696	85375	**50.33**

∗The object density of KiwiFruitLeaf is higher than that of existing datasets.

### 2.2 Methods

**Fig. 3:**
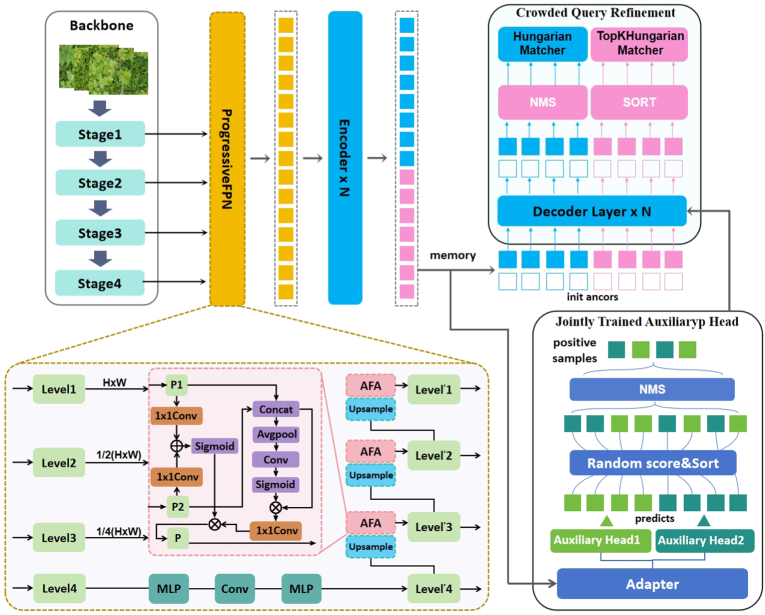
The overall framework of Leaf-DETR. It is equipped with ProgressiveFPN, CQR strategy and the improved JTAH. Through the extraction of discriminative features by P-FPN and the efficient training process equipped with CQR, the model is able to possess a powerful dense leaf detection capability.

For field dense leaf detection, we propose Leaf-DETR, a framework comprising the Progressive Feature Pyramid Network, Jointly Trained Auxiliary Head and Crowded Query Refinement strategy. To detect the dense leaves in the field, we propose the Leaf-DETR dense leaf detection framework, which includes the Progressive Feature Pyramid Network with a progressive framework, the Jointly Trained Auxiliary Head, and the Crowded Query Refinement strategy. As shown in Fig. 3, the input leaf image first undergoes preliminary feature extraction by the backbone. Subsequently, the multi-scale features extracted from the backbone further interact and fuse in the P-FPN, which enhances the distinguishability between individual leaves and adjacent leaves. Furthermore, these enhanced features are uniformly encoded by the encoder and then transmitted to the decoder and the jointly trained auxiliary head. The decoder receives information from both the encoder and the auxiliary head simultaneously to expand the training samples. It is equipped with a crowded query refinement strategy to mitigate the adverse effects caused by leaf overlap. Finally, the output results will retain the bounding boxes of both the intact leaves and the occluded leaves.

#### 2.2.1 Progressive Feature Pyramid Network

In the field scene, complex environmental factors significantly impact the effectiveness of leaf detection [44]. The mutual overlap of leaves confuses feature extraction, making it difficult to distinguish overlapping leaves. To this end, we propose a novel pyramid network - Progressive Feature Pyramid Network, equipped with a progressive architecture and an Adaptive Feature Aggregation module. Through multi-dimensional information extraction and the gradual fusion of features from adjacent levels, it enables the interaction between high-level and low-level information and clarifies the spatial relationship between individual leaves and adjacent leaves under dense vegetation.

**Progressive architecture.** The multi-scale features from the backbone contain the spatial information and texture information of the leaves. These pieces of information reveal the occlusion situation and texture features of the leaves. With the help of this information, the model's ability to distinguish the foreground and background of leaves can be enhanced. Therefore, we propose a progressive architecture for multi-scale information interaction. As illustrated in Fig. 3, it is designed as a feature fusion path from high to low, which enhances the texture information of the leaves at the lower levels with the spatial information of the leaves at the higher levels, thereby strengthening the distinguishability of the leaf edges and avoiding the confusion of the leaf features. Initially, it receives multi-scale feature maps from the backbone model. Among them, the feature map at the highest level undergoes feature mapping through a feed-forward neural network to generate the first output feature map, as described in equation (1):

(1) $$Level_{\mathrm{latest}}^{\prime }=MLP\left(Conv\left(MLP\left(Level_{\mathrm{latest}}\right)\right)\right),$$

where *MLP* represents the fully connected neural network layer, *Conv* represents the convolutional layer, *Level*_*latest*_ represents the input features of the highest layer, and $Level_{\mathrm{latest}}^{\prime }$ represents the output features of the highest layer. Subsequently, the high-level features carrying spatial information are upsampled by a factor of two, and are then input together with the adjacent low-level features carrying texture information into the Adaptive Feature Aggregation (AFA) module for feature fusion, resulting in low-level features that carry both spatial and texture information. Finally, multi-scale feature maps that integrate information at different scales are obtained. This process can be formally expressed as equation (2):

(2) $$Level_{i}^{\prime }=AFA\left(Level_{i},Upsample\left(Level_{i+1}^{\prime }\right)\right),$$

where *Level*_*i*_ represents the i-th level input feature, $Level_{i}^{\prime }$ represents the i-th level output feature, *AFA* represents the Adaptive Feature Aggregation module, and *Upsample* represents the upsampling operation.

**Adaptive Feature Aggregation.** Leaf overlap causes front-to-back occlusion, where both planar and interleaved leaf positions in images are key to judging adjacent leaf relationships. In view of the front-to-back occlusion relationship presented by the overlapping of leaves, both the planar position and the interlaced position of the leaves in the image are important factors for judging the relationship between adjacent leaves. By learning the positional relationships of the leaves, the model's ability to identify individual leaves can be improved. Therefore, the AFA module is designed as a dual-path structure capable of capturing spatial information and channel information, and with the help of this information, the model can better locate the leaves. Specifically, the input features $P_{1}\in R^{C\times H\times W}$ and $P_{2}\in R^{C\times H\times W}$ are processed to establish spatial dependencies among local feature mappings. *P*_1_ and *P*_2_ are then compressed into the global spatial information through a 1 × 1 convolution layer. A Sigmoid activation function is used to capture and enhance the prominent global spatial regions, and this information is utilized to calibrate the feature map, so as to obtain the planar positions of each leaf:

(3) $$F_{s}=Sigmoid\left(Conv\left(P_{1}\right)⊕Conv\left(P_{2}\right)\right),$$

where *F*_*s*_ represents the captured global spatial information, and the ⊕ sign represents the element-wise addition operation. In addition, the interlaced information of the leaves is contained in the channel features. In order to dynamically capture the global information, it is necessary to model the channel information [45]. Specifically, the features *P*_1_ and *P*_2_ are first concatenated along the channel dimension to form the feature $P_{1,2}\in R^{2C\times H\times W}$:

(4) $$P_{1,2}=Concat\left(P_{1},P_{2}\right),$$

subsequently, we use a cascaded module consisting of global average pooling, convolutional layer, and Sigmoid activation to extract the weight of the channels:

(5) $$P_{c}=Sigmoid\left(Conv\left(AvgPool\left(P_{1,2}\right)\right)\right),$$

where *AvgPool* represents the global average pooling operation and *P*_*c*_ represents the global channel weight. When extracting leaf features, the interlaced information is an important characteristic for determining whether there is an overlapping situation at the leaf edges. Therefore, we use the channel information as a guide to retain the important leaf edge features:

(6) $$F_{c}=Conv\left(P_{c}⊗P_{1,2}\right),$$

where *F*_*c*_ represents the captured global channel information, finally, *F*_*s*_ is multiplied with *F*_*c*_ to obtain the enhanced feature *P* that contains enhanced spatial location information,

(7) $$P=F_{c}⊗F_{s}.$$

#### 2.2.2 Crowded query refinement

Auxiliary training injects diverse positive samples into the network, enabling the model to better learn leaf morphological features and spatial distribution, and thus enhancing dense leaf region detection. Auxiliary training introduces a rich variety of positive samples into the network, helping the model better learn the morphological features and spatial distribution patterns of leaves, thereby improving its ability to detect dense leaf regions. However, these samples mainly optimize the decoder, while the backbone network and feature extraction layers prior to the decoder remain under-trained. This leads to missed detections during inference, especially in scenes with heavy leaf occlusion or complex backgrounds. To address this issue, we propose a crowded query refinement strategy, which introduces an additional branch to expand the initial queries of the decoder, generating more trainable queries and thus enhancing the model's coverage of potential leaf regions. To handle the increased matching cost caused by the extra queries, we adopt a one-to-many matching mechanism during training, combined with crowded candidate box cullingNMS to select the optimal detection results. This strategy effectively improves training efficiency and sample utilization, while maintaining model lightweightness, and enhances the robustness of detecting dense, occluded, or small-sized leaves.

Specifically, as shown in Fig. 3, first, the CQR strategy employs an additional parallel branch during the network training process to generate extra queries. Both these extra queries and the original queries are processed through the head of the encoder to produce two parts of the initial input for the decoder. Second, in the decoder stage, after the queries pass through the decoder layers, corresponding coordinate representations and classification scores are generated. Since each query can represent the location of a potential instance, crowded candidate box culling we apply class-agnostic NMS to the original queries to reduce similar queries for the same leaf. This, in turn, alleviates the computational burden of the Hungarian matcher and stabilizes the network training. Third, to increase the number of trainable query samples, crowded candidate box culling guides additional queries to be ranked based on their classification scores without applying non-maximum suppression (NMS), followed by matching them using the Top-K Hungarian matching algorithm. Third, to increase the trainable query samples, the extra queries are not processed with NMS. After a round of sorting according to the classification scores, the TopK Hungarian matching algorithm is used to match them. The TopK Hungarian matching algorithm has a one-to-many characteristic, which will bring a large number of selectable queries for objects to expand the trainable samples. Specifically, the Top-k Hungarian algorithm replicates each ground truth box k times. For each replica, once a predicted box is assigned, it is removed from the assignment pool, and the next replica assigns only among the remaining unassigned predictions. This procedure is repeated k times. By testing the performance with Top-k values ranging from 1 to 8, we determined that the model achieves its best performance when Top-k = 4; the corresponding results are provided in the Supplementary S.5. Therefore, the output of the decoder consists of two parts:

(8) $$Output=HM\left(NMS\left(Queries_{\mathrm{ori}}\right)\right)+TopKHM\left(Sort\left(Queries_{\mathrm{extra}}\right)\right),$$

where *Output* is the final matching result, *HM* is the Hungarian matching algorithm, *TopkHM* is the TopK Hungarian matching algorithm, *Queries*_*ori*_ are the original queries, and *Queries*_*extra*_ are the additional queries.

Due to the larger number of extra queries and the adoption of the one-to-many matching approach, we apply an extra loss function with a lower weight to these extra queries. This is done to prevent the accuracy from decreasing during the inference process as a result of the one-to-many matching. The loss function is defined as:

(9) $$Loss=Loss_{\mathrm{ori}}\left(O_{\mathrm{ori}}\right)+Loss_{\mathrm{extra}}\left(O_{\mathrm{extra}}\right),$$

where *Loss*_*ori*_ and *Loss*_*extra*_ represent the original and additional loss functions respectively, and *O*_*ori*_ and *O*_*extra*_ represent the original and additional queries respectively. These extra queries are used as a means of accelerating training and are activated during training while being disabled during testing. This strategy supplies abundant positive samples for network learning, accelerating training with negligible inference cost. This strategy provides the network with a large number of positive samples for learning, thereby accelerating the training process with only a slight increase in inference cost.

#### 2.2.3 Improved jointly trained auxiliary head

The one-to-one matching mechanism leads to insufficient positive samples and missed detections. The one-to-one matching mechanism of the end-to-end model leads to insufficient positive samples, which will cause the phenomenon of missed detection of leaves. Considering the diverse positions of leaves in the dense leaf detection task, we believe that introducing a one-to-many matching mechanism can alleviate this phenomenon [46]. Inspired by Refs. [10,47], we introduce the improved Jointly Trained Auxiliary Head. Auxiliary heads are single-stage or two-stage detection heads. These auxiliary heads are composed of single-stage or two-stage detection heads. Feature transformation is carried out through the Adapter and then input into the auxiliary head for self-training. Given the complex positions of the leaves, we believe that the easily detectable surface leaves with high confidence in the auxiliary head are insufficient to provide the model with diverse positive samples. Therefore, referring to the design of CQR, we discard the confidence values in the auxiliary head and instead assign randomized confidence values to each sample and use NMS to suppress the overlapping samples. These positive samples are embedded into position encoding and input into the decoder for training. This method enriches decoder positive samples to offset sparsity. This method can introduce diverse positive samples to the decoder, thus compensating for the problem of sparse positive samples in the decoder.

As illustrated in Fig. 3, the memory output from the encoder goes through a transformation process within the Adapter to align with the input specifications of the auxiliary heads. Following this adaptation, the auxiliary heads leverage the conventional one-to-many matching mechanism for self-training purposes, as formalized by the following equation:

(10) $$pos_{i}=AuxHead_{i}\left(Adapter\left(memory\right)\right),$$

where *memory* represents the output of the encoder, *AuxHead*_*i*_ represents the i-th auxiliary head and *pos*_*i*_ is the positive sample of *AuxHead*_*i*_. Subsequently, all positive samples are concatenated and randomly assigned confidence scores. After being suppressed by Non-Maximum Suppression (NMS), they are embedded as position encodings and fed into the decoder as supplementary positive training samples, as shown in the following equation:

(11) $$pos=NMS\left(concat\left(\overset{n}{\underset{i=1}{⋃}}pos_{i}\right),\left\{s_{j}|s_{j}∼U\left(0,1\right),j=1,\ldots ,m\right\}\right)$$

where *n* represents the number of auxiliary heads, *pos*_*i*_ represents the positive samples of the i-th head, *m* is the number of all positive samples, and U(0,1) represents the uniform distribution over the interval (0, 1).

#### 2.2.4 Evaluation metrics

Our work employs four principal metrics for rigorous multi-aspect performance quantification: Average Precision (*AP*), Precision, Recall, and *F*_1_ score. These criteria collectively evaluate detection reliability, completeness, and class-wise discriminative capacity through complementary analytical perspectives.

**Precision** quantifies prediction trustworthiness by measuring the proportion of true positives among all positive predictions:

(12) $$\text{Precision}=\frac{TP}{TP+FP}$$

where *TP* represents correctly identified objects and *FP* denotes background regions erroneously classified as objects. High precision indicates minimal false positive detections in network predictions.

**Recall** evaluates detection completeness through the ratio of correctly identified positives to all actual objects:

(13) $$\text{Recall}=\frac{TP}{TP+FN}$$

where *FN* corresponds to undetected true objects. Superior recall reflects an effective reduction of missed detections across test samples.

**Average Precision** **(*AP*)** encapsulates the precision-recall trade-off across confidence thresholds through numerical integration of the precision-recall curve (PRC). The computation protocol involves:

1. Confidence-score-based sorting of predictions per class;
2. Threshold-wise precision and recall calculation;
3. PRC construction with area-under-curve (AUC) computation:

(14) $$AP=\int_{0}^{1}P\left(R\right)\;dR$$

where *P*(*R*) represents precision as a recall-dependent function.

The **Mean Average Precision** **(*mAP*)** provides global performance assessment through class-wise *AP* averaging, establishing a unified benchmark for multi-category detection systems.

The detailed information about these metrics can be found in the appendix.

## 3 Results

### 3.1 Settings

The Leaf-DETR framework was trained on a single NVIDIA A30 GPU with a batch size of 2, using employs the AdamW optimizer with an initial learning rate of 2e-4 and a weight decay coefficient of 1e-4, implementing gradient clipping through L2-norm normalization constrained to a maximum threshold of 0.1. A stepwise learning rate scheduling strategy is activated at the 11th training epoch while maintaining ResNet-50 (R50) [48] as the fixed backbone architecture throughout the 12-epoch training cycle. The dataset undergoes stratified partitioning with an 8:2 training-validation split ratio, where the final weights from the 12th epoch are exclusively utilized for validation without implementing intermediate checkpoint selection. For comparative model architectures, parameter configurations specified in the original publications are strictly maintained, ensuring R50 backbone consistency across all models to ensure experimental comparability. All computational processes follow deterministic initialization protocols with fixed random seeds for reproducibility verification.

### 3.2 Comparative experiment of different models

#### 3.2.1 Quantitative comparison

To validate the effectiveness of our proposed network (Leaf-DETR) in the task of dense leaf detection, we compared it with several mainstream object detection models on the dense kiwifruit leaf object detection dataset, including two-stage detectors, one-stage detectors, and end-to-end detectors. To ensure fairness, all competing models are trained for an identical 12-epoch duration, leveraging a ResNet50 backbone and their respective originally published hyperparameter settings to guarantee a scrupulously fair comparative landscape, with detailed outcomes presented in Table 2. Furthermore, we conducted 10 independent experiments with different seeds to verify the stability of the experimental results, and these results can be found in Supplementary S.2.

**Table 2:** Comparison of different object detection models in kiwifruit leaf detection.

Model	Evaluation indicators				
	mAP	mAP@50	mAP@75	AR@100	AR@300
ATSS [49]	65.1%	85.2%	71.8%	70.0%	70.0%
Sabl [50]	65.3%	84.7%	72.0%	70.1%	70.1%
YOLOv5L	40.3%	72.6%	41.2%	46.4%	46.4%

Faster R-CNN [20]	63.9%	84.5%	70.9%	68.7%	68.7%
Cascade R-CNN [51]	63.2%	83.6%	70.5%	68.5%	68.5%
Libra R-CNN [52]	63.7%	84.4%	71.0%	68.4%	68.4%
Dynamic R-CNN [53]	56.7%	77.6%	63.1%	64.8%	64.8%

DeformableDETR_*5scales*_ [54]	46.7%	73.6%	52.2%	55.3%	55.3%
DINO_*5scales*_ [55]	62.7%	89.2%	74.0%	71.7%	77.7%
RT-DETR_*3scales*_ [56]	57.3%	79.6%	63.5%	62.7%	67.3%

DDQ_*5scales*_ [57]	66.4%	92.6%	78.1%	71.7%	78.3%
CrowdDet_*4scales*_ [58]	60.8%	89.6%	72.4%	66.2%	71.7%
GFL [59]	65.2%	88.4%	71.7%	70.0%	70.0%
DDOD [60]	65.0%	85.2%	71.8%	69.5%	69.5%

CO-DETR(baseline) [47]	66.3%	92.2%	77.6%	71.6%	78.1%
CO-DETR + P-FPN	66.5%	92.5%	78.1%	71.7%	79.4%
CO-DETR + CQR	66.7%	92.8%	78.6%	71.7%	79.3%
Leaf-DETR(ours)	**66.9**%	**93.2**%	**79.2**%	**71.9**%	**79.5**%

∗As divided by the horizontal line, they are categorized into single-stage detectors, two-stage detectors, end-to-end detectors, dense detectors and the ablation models of our method. Various components have provided performance improvements for the baseline, and ultimately, Leaf-DETR surpasses existing object detectors in all indicators, confirming its robust dense leaf detection capability.

First, the empirical data reveal that Leaf-DETR consistently surpasses its counterparts, achieving a discernible 0.6%0.5% uplift in mAP compared to the baseline, a testament to its refined overall detection capabilities. Moreover, this advancement is particularly pronounced in precision-centric metrics, with Leaf-DETR securing improvements of 1%0.6% in mAP@50 and 1.6%1.1% in mAP@75, indicating a superior faculty for precise bounding box delineation, especially for objects with substantial overlap. Significantly, the framework demonstrates a notable 1.4%1.2% enhancement in the average recall rate (AR@300), underscoring its augmented capacity to correctly identify a larger fraction of true leaf instances, a critical advantage in densely populated foliar environments where omissions are common. Consequently, these multifaceted performance gains unequivocally establish Leaf-DETR's preeminence, showcasing not merely an elevated leaf recall rate but also substantially improved bounding box localization accuracy and classification fidelity, thereby validating its profound robustness and superior efficacy in navigating the complexities inherent to dense leaf detection scenarios.

At the same time, we observe that AR@100 underperforms AR@300 in terms of performance improvement, and this gap needs to be clarified with additional experiments. At the same time, an observable discrepancy emerges wherein the performance uplift registered by the AR@100 metric substantially trails that achieved by AR@300, where this divergence necessitates a nuanced explication rooted in several critical factors. First, the primary constraint arises from the AR@100 evaluation protocol itself, which, by capping the maximum detectable instances at 100, inherently curtails the model's capacity to comprehensively enumerate all foliar targets, particularly when actual leaf counts in certain images exceed this threshold. Second, this restrictive ceiling not only impedes the full expression of the model's recall potential but also renders the AR@100 metric less sensitive for accurately gauging recall enhancements in scenarios characterized by high object density, including instances where foliar counts surpass the 100-object limit. Conversely, the AR@300 metric, by permitting a substantially larger quota of 300 potential detections, furnishes a more capacious evaluation headroom; this expanded allowance facilitates a more exhaustive prediction landscape, thereby affording considerable latitude for sophisticated anchor box optimization strategies and accommodating inherent inference redundancies, as corroborated by Ref. [61]. Indeed, this operational paradigm, where predictive capacity significantly exceeds the typical object count per image, resonates with established practices, such as the deployment of 100 queries in DETR architectures for datasets like COCO, where instance density is comparatively sparse, thereby ensuring robust detection across varying object numbers. Hence, within the specific context of dense object detection paradigms, particularly those characterized by high instance numbers per image as in our study, the AR@300 metric unequivocally offers a more veracious and comprehensive assessment of recall performance, rendering it a superior evaluative instrument over the more constrained AR@100.

In addition, after research, YOLOv5 performs poorly in dense object detection primarily because its backbone uses 32 × downsampling, causing tiny objects to lose most or all of their information in deep feature maps. Additionally, its grid-centered assignment strategy tends to miss or misidentify objects when multiple centers fall into the same grid cell under crowded conditions. Therefore, YOLOv5 is better suited for scenarios with larger, sparsely distributed objects and a high demand for inference speed, such as road vehicle detection or pedestrian recognition against simple backgrounds.

**Fig. 4:**
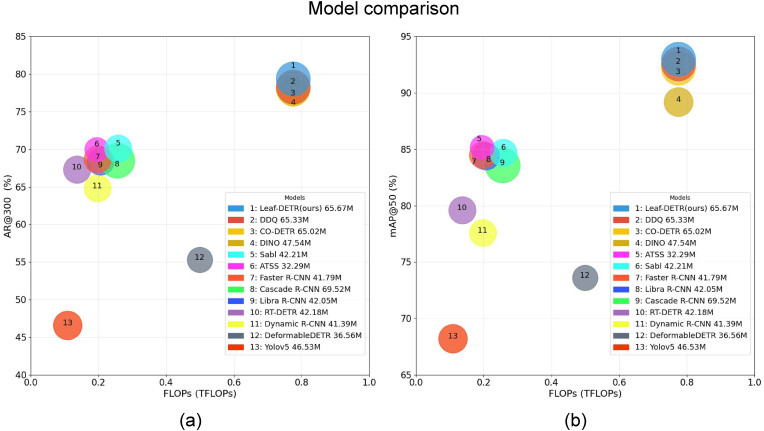
Detection performance comparison. (a) The relationship between mAP@50 and FLOPs (T). (b) The relationship between AR@300 and FLOPs (T). Leaf-DETR stands out by achieving the collaborative optimization of precision and recall. Leaf-DETR achieves an excellent balance between computational efficiency and detection performance, demonstrating more outstanding detection results compared to similar models.

To clarify the interplay between computational overhead and detection efficiency, we present a detailed visualization of the key performance metrics as shown in Fig. 4. This graphical representation employs a 3D scatter plot, where mAP@50 and AR@300 occupy the orthogonal axes, TFLOPs are mapped to the horizontal axis, and the magnitude of the model parameters is scaled to represent the bubble size. Firstly, the problem of high inter-leaf occlusion rate prevalent in dense leaf detection tasks poses great challenges, often leading conventional detectors to prioritize identifying unoccluded surface leaves while ignoring partially occluded samples. In the figure, Leaf-DETR stands out by achieving a collaborative optimization of accuracy and recall, demonstrating an improvement of 0.5% in mAP and 1.2% in AR@300, with only a marginal parameter increase over the state-of-the-art baseline. Thus, not only are more leaves detected, but also higher fidelity bounding-box localization is provided, which is essential for powerful downstream agricultural analysis.

#### 3.2.2 Qualitative comparison

**Fig. 5:**
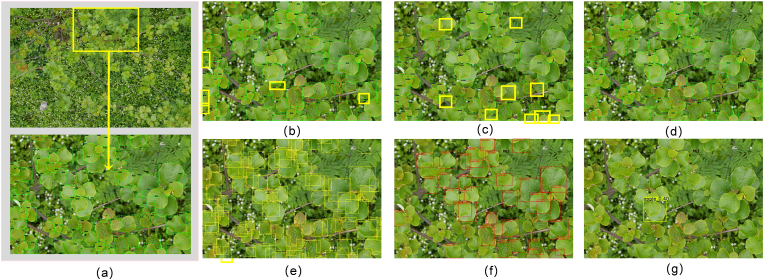
Visualization comparison. (a)Select the image area with dense leaves and the result of Leaf-DETR. (b) SABL. (c) Faster R-CNN. (d) DDQ. (e) T-Rex. (f) Grounding-DINO. (g) YOLO-World. The yellow boxes indicate undetected leaves. The yellow boxes represent the undetected leaves, and Leaf-DETR achieves comprehensive detection. Miss rates for the evaluated models are: (a) 4.5%, (b) 12.9%, (c) 14.0%, and (d) 7.1%. Miss rate is not computed for the multimodal model. The yellow boxes represent the undetected leaves. Compared with other models, Leaf-DETR has no cases of missed detection, confirming its superior detection coverage.

To intuitively demonstrate the improvements brought by our proposed model and dataset to dense image detection tasks. To intuitively corroborate the efficacy and precision of our proposed model within dense image detection tasks, a representative dense image is selected for a comparative visualization of inference results from four distinct architectures: Sabl, Faster R-CNN, DDQ, and Leaf-DETR. Each comparative model utilizes its optimal parameter configuration as determined by the mAP@50 metric, with detailed visual comparisons systematically presented in Fig. 5. Visual inspection, as depicted, reveals that all evaluated models exhibit, to varying degrees, omissions of certain leaves; however, commendably, instances of erroneous detections such as duplicate annotations or false positives are largely absent. Notably, Leaf-DETR demonstrates exceptional detection performance when confronted with highly challenging, occluded edge regions, proficiently identifying and precisely delineating overlapping foliage, multi-scale leaves, and peripheral samples frequently overlooked by alternative methodologies. This superior capability is fundamentally attributed to our adopted cross-scale feature optimization strategy, which markedly enhances the model's proficiency in discerning and extracting target features within visually complex milieus. Consequently, these empirical findings robustly substantiate the enhanced efficacy of our architecture in adeptly addressing the inherent detection challenges posed by severe leaf occlusion, thereby underscoring its significant practical utility in complex agricultural imaging applications.

#### 3.2.3 Comparison with multimodal models

Given the rapid development of multimodal detection architectures, state-of-the-art multimodal systems demonstrate superior performance in general detection domains, particularly T-Rex [62], Ground-DINO [63], and YOLO-World [64]. Given the rapid development of multimodal detection architectures, Leaf-DETR is ranked alongside well-known contemporary multimodal systems, in particular, T-Rex [62], Ground-DINO [63] and YOLO-World [64]. Multimodal models typically require extensive multimodal data to provide comprehensive knowledge for general-purpose detection. However, domain-specific datasets are often scarce, with complex data acquisition processes and incomplete modality coverage. In high-complexity dense detection tasks, the design of general-purpose detectors fails to adapt to the unique detection patterns of specialized domains. To validate the necessity of our work, we conduct comparative experiments between Leaf-DETR and mainstream multimodal detectors. This comparative study aims to evaluate the necessity of task-specific datasets and model design, rather than emphasizing performance superiority. ItThis comparison experiment strictly follows the same protocol, where T-Rex uses visual cues for object localization, while Ground-DINO and YOLO-World rely on text cues for open vocabulary detection mechanisms. The empirical results, shown in Fig. 5, clearly show that Grounding-DINO and YOLO-World consistently fail to detect effectively in dense scenes and will miss most of the detected objects. On the contrary, thanks to its architecture tailored for dense detection, T-Rex performs remarkably well on the dense leaf detection task, successfully localizing the majority of leaf instances; On the contrary, T-Rex shows a commendable degree for the intensive leaf detection task, successfully locating most leaf instances; Leaf-DETR exploits the efficacy of the aforementioned architectural innovations to significantly outperform other methods by detecting challenging, surrounding occluded, and overlapping leaves. Thus, this systematic evaluation confirms that for precision agriculture applications requiring robust dense object localization and fine semantic understanding, specialized detection frameworks such as Leaf-DETR maintain enduring applicability and maintain provable superiority over generalized multimodal paradigms.

### 3.3 Ablation study

To analyze the independent contribution and collaborative efficacy of our proposed architecture enhancement modules, we systematically conduct comprehensive ablation experiments, especially focusing on P-FPN and CQR. Since we regard the improved JTAH as an extension of the CQR strategy, our baseline is the CO-DETR model which is not equipped with the improvements of JTAH. The detailed quantification results of these ablation studies have been precisely summarized in Table 2, providing a refined assessment of the impact of each component. Empirical evidence derived from these studies clearly shows that both the CQR strategy and the P-FPN module make a discernible positive contribution to the overall performance of the dense kiwi leaf detection benchmark. Specifically, the Leaf-DETR framework integrating P-FPN and CQR outperforms the baseline CO-DETR by 0.5% in mAP, and achieves an improvement of 0.6% and 1.1% on AP@50 and AP@75, respectively. This benefits from the fine-grained features provided by the progressive pyramid structure, which provides a more refined feature representation for the regression of positioning boxes. Furthermore, this performance improvement also extends to recall, where our full method achieves a 1.2% gain on AR@300, indicating that the CQR strategy can effectively improve the performance of dense prediction models. Thus, these ablation results consistently validate the respective and common importance of P-FPN and CQR components, confirming their critical role in improving the bounding box localization accuracy and overall detection rate of the Leaf-DETR framework for dense object detection tasks.

#### 3.3.1 Validity verification of module

**Fig. 6:**
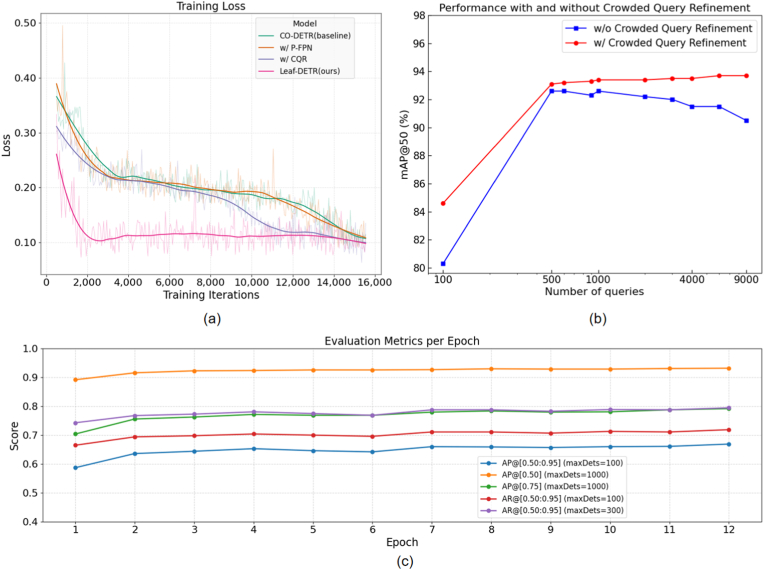
Training performance of different combinations. (a) The model equipped with CQR converges faster in the middle and late stages of training, while the model with both P-FPN and CQR demonstrates excellent training efficiency. (b) After being equipped with CQR, the model's performance in handling large-scale queries has been significantly improved. (c)The model equipped with CQR training metrics across epochs.

To empirically assess the purported stabilizing influence of our methodological refinements on the model training dynamics, a comparative analysis of training loss trajectories is conducted across models incorporating different components, with the results visually depicted in Fig. 6(a) and (c). Scrutiny of the convergence patterns reveals that while the orange and blue lines, representing baseline with CQR and baseline with P-FPN (which includes AFA) respectively, exhibit broadly similar convergence characteristics, the green line (baseline CO-DETR) demonstrates a comparatively slower convergence, particularly in the initial and terminal training phases. In striking contrast, the pink line, corresponding to our full Leaf-DETR, showcases a markedly accelerated convergence velocity relative to all other configurations, underscoring the substantial role of the CQR strategy in both stabilizing the training process and expediting convergence. Note that the curves are plotted against iterations, resulting in significant fluctuations in local loss values (dashed), whereas the global loss values (solid lines) consistently exhibit a converging trend. Furthermore, the integration of P-FPN, which inherently includes the AFA module, contributes to this rapid stabilization, with the complete Leaf-DETR model achieving stable convergence around the 3000-iteration mark. This expedited learning trajectory signifies that our proposed framework can attain a state of sufficient learning with a considerably reduced training quantum, thereby offering pronounced advantages in terms of computational resource efficiency and training time.

#### 3.3.2 Validity verification of CQR

To further explore how dense queries optimize the model, we compare the baseline and our proposed model, To further investigate the optimization impact of dense queries on the model, we conduct a comparison between the baseline model and our proposed model, as illustrated in Fig. 6(b). This investigation reveals that as the number of queries escalates, both CO-DETR and Leaf-DETR initially exhibit a concordant trend of performance enhancement. However, a critical divergence emerges when the query count approaches approximately 600; at this juncture, the standard CO-DETR, lacking the CQR mechanism, encounters a substantial training encumbrance stemming from the proliferation of highly similar queries, which markedly diminishes the computational efficiency of the Hungarian matching algorithm. In stark contrast, CO-DETR augmented with the CQR strategy sustains stable performance despite the escalating query volume, a resilience directly attributable to CQR's iterative query refinement capability. This iterative refinement process systematically discards redundant queries that correspond to the same instance at each stage, thereby alleviating the inferential burden on the model. Consequently, the CQR-equipped architecture adeptly maintains performance stability even amidst rapid query growth, highlighting the crucial role of dynamic query culling in managing computational demands and preserving efficacy in crowded query regimes.

### 3.4 Generalization experiment

**Fig. 7:**
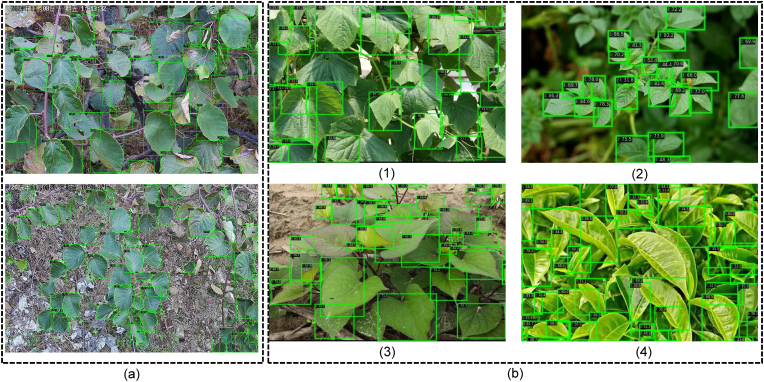
Detection results of images captured by monitoring devices and other crop images. (a) Images captured by monitoring devices. (b-1) Cucumber. (b-2) Potato. (b-3) Sweet Potato. (b-4) Tea. Our method demonstrates reliable generalization performance in the migration of application scenarios.

To evaluate Leaf-DETR's real-world applicability and generalization, we test it on surveillance-captured images outside the training dataset; quantitative and qualitative results are in Table 3 and Fig. 7(a). To evaluate the real-world applicability and generalization prowess of Leaf-DETR, images captured by operational surveillance equipment, distinct from the training dataset, are employed as novel input, with quantitative and qualitative results presented in Table 3 and Fig. 7(a) respectively. This evaluation is particularly challenging as the model is not exposed to data from this specific scene during its training phase. Moreover, a six-month temporal disparity between the original dataset collection and the surveillance image acquisition meant the kiwifruit leaves had progressed significantly through their growth cycle, manifesting widespread instances of withered and diseased foliage. These substantial alterations in leaf morphology and surface characteristics introduced considerable domain shift, inherently complicating accurate detection for any model. Despite these pronounced deviations—including variations in shooting angles and ambient lighting conditions relative to the training data—Leaf-DETR demonstrated remarkable robustness, successfully identifying over 90% of the leaves within the surveillance images, encompassing both front-facing and obliquely oriented specimens.

**Table 3:** Comparison of the generalization performance of surveillance images.

Model	Evaluation indicators				
	mAP	mAP@50	mAP@75	AR@100	AR@300
Sabl	36.6%	52.9%	40.5%	44.7%	44.7%
Faster R-CNN	28.8%	43.5%	31.8%	33.9%	33.9%
DDQ_5scales_	45.6%	70.1%	51.7%	58.6%	63.8%
CO-DETR_*5scales*_	37.9%	59.0%	43.5%	47.9%	54.4%
Leaf-DETR(ours)_5scales_	**50.8%**	**74.5%**	**58.7%**	**61.6%**	**67.8%**

∗The indicators of Leaf-DETR are higher than those of existing models, reflecting its powerful generalization performance.

Furthermore, to explore broader applicability, our approach was subsequently tested on four globally prevalent cultivated crops, as illustrated in Fig. 7(b) and Table 4, where it unexpectedly exhibited commendable detection performance on these diverse plant species even without species-specific training, its recall performance is comparable to that on kiwifruit leaves, suggesting strong baseline generalization capability. even without species-specific training, hinting at a strong foundational generalization. Consequently, these findings collectively underscore the robust generalization capabilities of Leaf-DETR, suggesting its potential for direct deployment in varied field environments and indicating significant promise for enhanced cross-crop detection accuracy with targeted fine-tuning.

**Table 4:** Comparison of the generalization performance of other crops images.

Crop	Evaluation indicators				
	mAP	mAP@50	mAP@75	AR@100	AR@300
Cucumber	37.8%	67.8%	38.8%	53.5%	58.1%
Potato	38.3%	67.1%	38.9%	56.5%	61.8%
Sweet Potato	34.8%	71.4%	27.5%	51.5%	56.1%
Tea	33.9%	61.4%	36.2%	53.6%	59.2%

## 4 Discussion

Leaf-DETR exhibits strong generalization performance, yet fine-tuning according to leaf growth stages remains essential for practical monitoring. Plant leaves typically progress through a life cycle that begins with emergence, continues through expansion, and ends in senescence. During this process, significant changes occur in size, shape, texture, and phenotypic appearance under disease stress. These variations make it difficult for a single model to effectively cover the entire growth cycle. In real-world deployment, the model must therefore be fine-tuned for specific growth stages to adapt to these evolving characteristics. Leaf-DETR's faster training convergence enables more rapid adaptation, supporting timely and accurate monitoring across different phases of leaf development. Notably, leaf phenotypic changes occur on the scale of days rather than milliseconds. Consequently, the emphasis on millisecond-level inference speed in real-time object detection has limited relevance here.

Although Leaf-DETR demonstrates excellent performance in dense leaf detection tasks, several challenges remain for its practical deployment. First, the model adopts a binary classification strategy—distinguishing only between background and leaves—rather than directly identifying leaf species or disease types. This design stems from inherent limitations in agricultural datasets: diseased leaves are often in early stages with subtle phenotypic symptoms that are difficult to recognize, or the disease has already spread extensively, resulting in severe scarcity of healthy leaf samples (in some datasets, healthy samples account for an extremely low proportion). These issues lead to extreme class imbalance, making multi-class classification impractical. Nevertheless, this simplified approach highlights the model's strong generalization capability: without species-specific fine-tuning, it achieves over 60% mAP@0.5 in leaf detection, indicating its potential as a foundational detector in multi-stage agricultural monitoring systems.

At the same time, the model exhibits limitations in handling non-orthographic views. In mountainous farming environments, where cameras often capture images at oblique angles rather than vertical perspectives, leaf shapes suffer from perspective distortion. On such angled monitoring images, mAP drops by 16.1%. The primary reason is the lack of data: leaf appearances under oblique angles exhibit substantial variations, posing a significant challenge to the model's generalization capability. If fine-tuned with additional data containing oblique-angle leaves, the detection performance would further improve. However, it is worth noting that AR@300 remains as high as 67.8%, suggesting that future improvements through synthetic viewpoint augmentation and multi-view fusion strategies could effectively mitigate this limitation.

Furthermore, in extremely dense scenarios such as tea plantations—where leaf density is 2–3 times higher than in kiwifruit orchards and leaf morphology differs significantly—many predicted bounding boxes fail to fully enclose entire leaves. Yet, numerous individual leaves exhibiting pronounced illumination differences between their two halves are still accurately detected, demonstrating the P-FPN module's ability to preserve fine-grained, low-level edge features. This extremely dense application scenario is even more challenging than kiwifruit leaf detection and imposes higher demands on detection density, requiring an increased number of initial anchor boxes or queries. However, a large number of such anchors or queries also leads to significantly higher computational costs. In fact, the CQR strategy is specifically designed to address these high-density scenarios by reducing model resource consumption and accelerating training. It should be noted that bounding box localization accuracy primarily relies on the model's ability to finely model image features, while high recall depends critically on the design of label assignment strategies and loss functions. In other words, in more demanding tasks, further performance gains cannot be achieved by a single module alone; they require holistic architectural co-optimization, such as integrating components like P-FPN. Within this context, the role of the CQR strategy is primarily to guide efficient model learning rather than directly improving final detection accuracy. Therefore, future work should continue to focus on enhancing detection performance under diverse conditions involving complex morphologies, varying viewing angles, and significant lighting variations.

## 5 Conclusion

In this study, we propose Leaf-DETR, a novel dense leaf detection framework that effectively addresses the challenges of feature confusion and poor network convergence in dense leaf detection scenarios through the introduction of a Progressive Feature Fusion Pyramid Network (P-FPN) and a Crowded Query Refinement (CQR) strategy. We also present kiwifruitleaf—the largest dense leaf detection dataset to date—comprising 1696 images with 85,375 annotated bounding boxes, on which our model achieves state-of-the-art performance.

Extensive evaluations demonstrate that Leaf-DETR not only delivers higher accuracy and recall but also exhibits strong generalization across different crop types and agricultural environments, maintaining robust performance under challenging conditions commonly encountered in real-world agriculture, such as detection on leaves of other crops and under non-vertical viewing angles. The construction of this high-density, high-overlap dataset provides a valuable resource for future research in agricultural computer vision, while the design of Leaf-DETR offers an effective solution to the feature confusion problem through P-FPN and addresses the optimization of densely packed bounding boxes via CQR, significantly accelerating network convergence. These contributions collectively advance the field by enabling more accurate leaf detection in complex field settings, which is essential for effective crop health assessment, disease detection, yield prediction, and precision agriculture applications. Future work will focus on further improving the model's performance in extreme scenarios through advanced data augmentation techniques and exploring the integration of Leaf-DETR with downstream agricultural tasks to build more comprehensive and practical field monitoring systems.

## Authors’ contributions

X.W.: Methodology, Software, Investigation, Writing – Original Draft. Y.W.: Formal Analysis and Writing – Review & Editing. X.D.: Data Curation and Writing – Review & Editing. L.Q.: Investigation. X.L.: Writing. P.Y. Supervised. L.L: revision. C.S.: Resources. A.S.: Data Curation. J.J.: Funding Acquisition. Q.W.: Conceptualization, Writing – Original Draft, Writing – Review & Editing, Supervision.

## Funding

This research was supported by the 10.13039/501100001809National Natural Science Foundation of China (No. 62506089), Guizhou Provincial Science and Technology Projects ([2024]002, CXTD[2023]027), Scientific and Technological Innovation Platform Research Project of Guizhou Province (CXPTXM[2025]024, CXPTXM[2025]026), Guizhou Province Youth Science and Technology Talent Project ([2024]317), and the Postgraduate Research Fund of Guizhou Province (2024YJSKYJJ090).

## Data availability

The code and detailed information are available at http://leafdetr.samlab.cn. For testing purposes, detailed instructions for running the model can be found in the repository's README file. Any further data or materials not included in the main text or supplementary files can be requested from the corresponding author.

## Declaration of competing interest

The authors declare that they have no known competing financial interests or personal relationships that could have appeared to influence the work reported in this paper.

## Footnotes

1 The Hungarian matching algorithm employs minimum-weight bipartite matching with a time complexity of *O*(*n*^3^), which becomes computationally expensive when n is large.
