# Supplementary material for: Leaf-DETR: Progressive adaptive network with lower matching cost for dense leaves detection
Source: Plant Phenomics. 2026 Feb 17;8(1):100182. doi: 10.1016/j.plaphe.2026.100182 (PMC13109562; doi:10.1016/j.plaphe.2026.100182)
Supplement: Multimedia component 1 [file mmc1.pdf]

Leaf-Detr: Progressive Adaptive Network with Lower Matching Cost for Dense Leaves Detection

Xiaoyang Wan[a], Yuxiang Wang[a], Xinyu Dong[a], Lufu Qin[a], Xixuan Luo[a], Peijia Yu[a,b], Liang Lei[b], Chao Sun[c], ASA Salgadoe[d], Jingjing Jiang[c,*], Qi Wang[a,*]

[a]*State Key Laboratory of Public Big Data College of Computer Science and Technology Guizhou University China*
[b]*the School Of Physics $ {\And }$ Optoelectronic Engineering Guangdong University of Technology Guangzhou 510006 China*
[c]*Suninfinit Agriculture Group Co. Ltd Shenzhen China*
[d]*Department of Horticulture and Landscape Gardening Faculty of Agriculture and Plantation Management Wayamba University Sri Lanka*

S.1. Metric definitions

All metrics used in the experiments, along with their definitions and formulas, are provided in Table S.1.

Table S.1: Definition of evaluation metrics.

Metric	Meaning	Formula
TP	Number of correctly identified target objects	–
FN	Number of undetected real target objects	–
FP	Number of background regions misclassified as target objects	–
Precision	The proportion of samples predicted as positive that are actually positive	$\text{Precision} = \frac{\text{TP}}{\text{TP} + \text{FP}}$
Recall	The proportion of correctly identified positive samples to all actual positive samples	$\text{Recall} = \frac{\text{TP}}{\text{TP} + \text{FN}}$
AP	Area under the precision-recall curve calculated at different confidence thresholds	$\text{AP} = \int_0^1 P(R)\, dR$
AP@n	Area under the precision-recall curve at confidence threshold n	$\text{AP} = \int_0^n P(R)\, dR$

S.2. Multiple seed experiments

To verify the reproducibility of our results, we independently ran the model 10 times under identical experimental settings using different random seeds, and computed the mean of each metric (denoted as the sample mean). These means were compared against the main result reported in the paper (denoted as H_0) using a one-sample t-test.Experimental results are shown in Table S.3 and Table S.2.The results show that mAP, AR@100, and AR@300 all yield p-values greater than 0.05, indicating no statistically significant difference between the repeated runs and H_0. For AP@50 and AP@75, the p-values are below 0.001 due to extremely low variance across the 10 runs; however, the absolute deviations are only −0.0025 and −0.0060, respectively—reflecting minor systematic offsets rather than meaningful performance degradation.

*Corresponding author

Table S.2: Performance across 10 random seeds.

Seed	mAP	mAP@50	mAP@75	AR@100	AR@300
1	66.8%	92.9%	78.7%	71.9%	79.6%
2	67.1%	93.0%	78.8%	71.9%	79.5%
3	66.9%	92.9%	78.6%	71.9%	79.6%
4	66.8%	93.0%	78.6%	71.8%	79.4%
5	66.8%	93.0%	78.6%	71.9%	79.5%
6	66.9%	92.9%	78.7%	71.9%	79.5%
7	66.8%	92.9%	78.7%	71.9%	79.4%
8	66.8%	92.9%	78.2%	71.8%	79.3%
9	67.0%	93.0%	78.6%	71.9%	79.5%
10	66.7%	93.0%	78.5%	71.8%	79.4%

Table S.3: Statistical analysis of repeated experiments.

Metric	Sample mean	H_0 Mean	p-value
mAP	66.86%	66.9%	0.3092
AP@50	92.95%	93.2%	<0.001
AP@75	78.60%	79.2%	<0.001
AR@100	71.87%	71.9%	0.0811
AR@300	79.47%	79.5%	0.3434

S.3. Anchor box scale distribution

To select the parameters of the sliding window method, we conducted a distribution analysis on the Groundingtruth of the proposed dataset, as shown in Figure S.1. According to the distribution, leaf bounding boxes have widths and heights not exceeding 400 pixels, with aspect ratios (width-to-height) generally close to 1. The sliding window is configured with an overlap ratio of 21% in the width direction and 47% in the height direction, which is sufficient to cover leaves of all scales. Since this configuration ensures complete coverage of leaf-occupied regions and effectively prevents missed detections, post-processing for bounding box merging is unnecessary.

S.4. Model configuration details

The annotation model employs the Deformable DETR architecture from the official MMDetection configuration, optimized with the AdamW optimizer using an initial learning rate of 2e-4 and a weight decay coefficient of 1e-4. Gradient clipping is applied via L2 norm normalization with a maximum threshold of 0.1. A step-wise learning rate scheduling strategy is activated starting from the 11th training epoch. Throughout the entire 12-epoch training schedule, ResNet-50 (R50) is used as a fixed backbone network without fine-tuning. The dataset is partitioned hierarchically into training and validation sets at an 8:2 ratio. Only the final model weights from the 12th epoch are used for evaluation, without selecting intermediate checkpoints. For all baseline and comparative model architectures, hyperparameters strictly follow the specifications reported in their original publications. All experiments adhere to a deterministic initialization protocol and utilize a fixed random seed to ensure reproducibility.

S.5. Selection of Top-k value

To select the key parameter Topk value of the Topk Hungarian algorithm, we conducted 8 groups of experiments, setting Topk from 1 to 8 respectively. The results of these experiments are shown in Table S.4. Based on the experimental results, we selected Topk=4 as the final number of replications. However, we found that the performance continues to improve when Topk is between 1 and 4, but continues to decline when Topk is between 5 and 8. After analysis, we believe that the reason for the performance decline is that when a Groundingtruth is matched with too

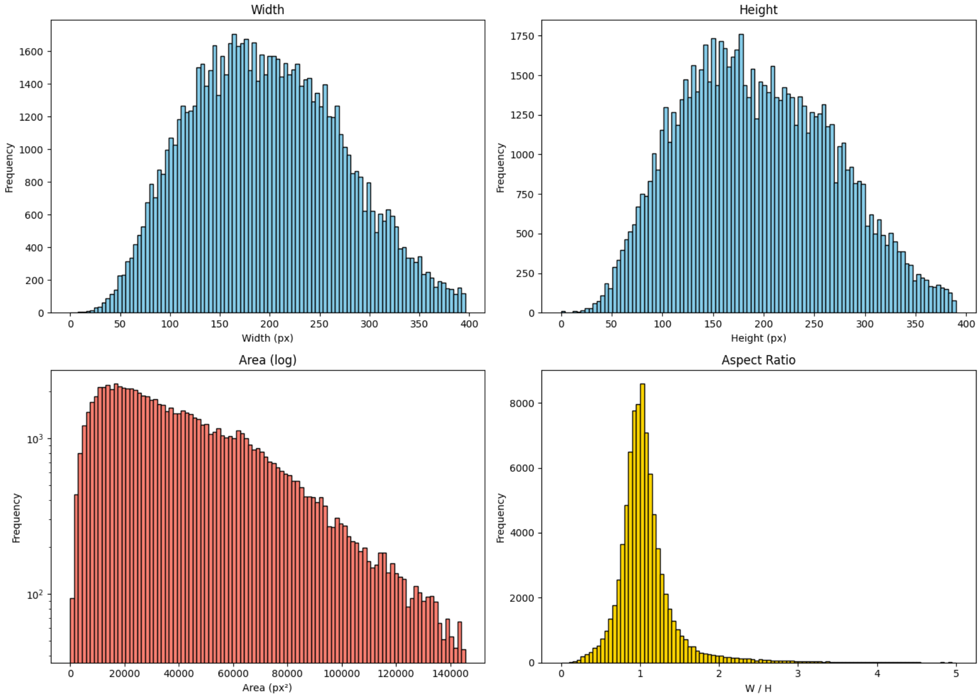

Figure S.1: Scale distribution of the anchor box.

many boxes, low-quality predicted boxes will be assigned. Low-quality predicted boxes often have defects in localization or IoU, which leads to negative training phenomena during network training, that is, the network learns worse samples. Moreover, as the number of Topk continues to increase, the efficiency of network training will continue to decline. Therefore, an excessively high Topk will not bring higher performance.

Table S.4: Performance with different Top-k values.

**Topk**	**mAP**	**mAP@50**	**mAP@75**	**AR@100**	**AR@300**
1	66.3%	92.4%	77.7%	71.6%	78.1%
2	66.4%	92.6%	77.7%	71.7%	78.6%
3	66.7%	92.9%	78.6%	71.7%	79.3%
4	66.9%	93.2%	79.2%	71.9%	79.5%
5	66.8%	93.1%	79.1%	71.9%	79.4%
6	66.6%	92.9%	78.7%	71.9%	79.4%
7	66.6%	92.6%	78.7%	71.9%	78.9%
8	66.4%	92.5%	78.3%	71.5%	78.5%
